# The intersection between space or ‘dark nature’, and people living with mild cognitive impairment or dementia: a mixed‐methods survey study

**DOI:** 10.1002/alz70858_105398

**Published:** 2025-12-26

**Authors:** Harmony Jiang, Paul McCrone, Catherine Carr, Charlotte Stoner

**Affiliations:** ^1^ University of Greenwich, London, London, United Kingdom; ^2^ Queen Mary University of London, London, London, United Kingdom

## Abstract

**Background:**

Interacting with or contemplating space or ‘dark nature’ can increase wellbeing through mechanisms such as increasing feelings of awe or wonderment. However, this has not been explored for people living with mild cognitive impairment or dementia in previous published research.

**Method:**

53 participants (56‐90 years old, 39.6% female) with a self‐reported diagnosis of mild cognitive impairment or dementia completed a one‐off online/remote mixed‐methods survey asking about their demographics, and views and experiences of space or ‘dark nature’, and space‐related activities. Qualitative data was analysed via thematic analysis and quantitative data was analysed using Excel.

**Result:**

Participants described space through physical and psychological/emotional/social aspects. Thinking about space and our place in the universe elicited feelings of awe for some participants. Participants were also concerned about space littering and preserving space. Though some participants described space as being mysterious and not fully knowing ‘what is up there’, others expressed excitement or contentment. Some participants felt more negative feelings such as anxiety, frustration and shame when thinking about space. Most of the participants (58.5%) said they would go stargazing now and 60.4% said they would attend a talk on space. Facilitators for space‐related activities included an easy to travel to and accessible location, option to join remotely and low cost.

**Conclusion:**

This study provides novel insights into how people living with mild cognitive impairment or dementia conceptualise space or ‘dark nature’, and the universe. It suggests potential mechanisms of action for the positive effects of contemplating space or carrying out space‐related activities, and barriers and facilitators to space‐related activities. This could help future researchers develop space‐related or ‘dark nature‐based’ interventions tailored for this population.

